# Perceived Stigma of Sudden Bereavement as a Risk Factor for Suicidal Thoughts and Suicide Attempt: Analysis of British Cross-Sectional Survey Data on 3387 Young Bereaved Adults

**DOI:** 10.3390/ijerph14030286

**Published:** 2017-03-09

**Authors:** Alexandra Pitman, Khadija Rantell, Louise Marston, Michael King, David Osborn

**Affiliations:** 1 UCL Division of Psychiatry, University College London, London W1T 7NF, UK; michael.king@ucl.ac.uk (M.K.); d.osborn@ucl.ac.uk (D.O.); 2 Camden and Islington NHS Foundation Trust, London NW1 0PE, UK; 3 Education Unit, UCL Institute of Neurology, University College London, London WC1N 3BG, UK; k.rantell@ucl.ac.uk; 4 UCL Department of Primary Care and Population Health, University College London, London NW3 2PF, UK; l.marston@ucl.ac.uk

**Keywords:** suicide, self-harm, bereavement, stigma, depression, support, risk factor

## Abstract

The sudden death of a friend or relative, particularly by suicide, is a risk factor for suicide. People who experience sudden bereavement report feeling highly stigmatised by the loss, potentially influencing access to support. We assessed whether perceived stigma following sudden bereavement is associated with suicidal thoughts and suicide attempt. We analysed cross-sectional survey data on 3387 young adults bereaved by the sudden death of a close contact. We tested the association of high versus low perceived stigma (on the stigma sub-scale of the Grief Experience Questionnaire) with post-bereavement suicidal ideation and suicide attempt, using random effects logistic regression, adjusting for socio-demographic factors, pre-bereavement psychopathology, and mode of sudden bereavement (natural causes/unnatural causes/suicide). Subjects with high perceived stigma scores were significantly more likely to report post-bereavement suicidal thoughts (adjusted odds ratio (AOR) = 2.74; 95% confidence interval (CI) = 1.93–3.89) and suicide attempt (AOR = 2.73; 95% CI = 2.33–3.18) than those with low stigma scores. People who feel highly stigmatised by a sudden bereavement are at increased risk of suicidal thoughts and suicide attempt, even taking into account prior suicidal behaviour. General practitioners, bereavement counsellors, and others who support people bereaved suddenly, should consider inquiring about perceived stigma, mental wellbeing, and suicidal thoughts, and directing them to appropriate sources of support.

## 1. Introduction

The search for modifiable risk factors for suicide underpins the suicide prevention research agenda. Sudden bereavement, particularly by suicide [1], is now recognised as a robust risk factor for suicide [2], but explanations for this are unclear. Studies controlling for mental illness indicate that neither heritability [2,3] nor assortative mating [4] completely account for the observed association. An alternative explanation is perceived stigma; the subjective awareness of others’ negative attitudes [5]. This is a common feature of sudden or violent bereavements and may influence access to support [1]. Stigma is also potentially modifiable [6]. Studies comparing grief reactions after different causes of death reveal that experiences of stigma, shame, and concealing the cause are reported after all modes of bereavement, but particularly after violent deaths [1,7] and specifically suicide [8]. Accounting for high levels of perceived stigma has been found to attenuate the association of suicide bereavement with suicide attempt [9], suggesting its role as a mediator of suicide risk. The implication is that anti-stigma interventions might reduce the risk of suicide attempt in people who experience sudden bereavement, perhaps by reducing distress and/or optimising support. 

The means by which stigma creates barriers to help-seeking have been well-described in relation to mental illness [10], but less well in relation to sudden bereavement [7,11,12,13]. In people with mental illness, stigma is hypothesised to contribute to suicidality through factors such as social isolation, hopelessness, and a perception of being a burden [14]. The same might be theorised after sudden bereavement, when avoidance might arise due to embarrassment, or fear of appearing socially incompetent [15]. Feeling stigmatised by a death contributes to a sense of thwarted belongingness and poor social support; both of which may engender suicidal thoughts [16]. Our objective was to investigate whether high levels of perceived stigma after sudden bereavement are associated with suicidal behaviour. To do this, we analysed British cross-sectional survey data on adults who had experienced sudden bereavement. Our hypothesis was that high stigma scores are associated with post-bereavement suicidal behaviour and depression. To build our understanding of mechanisms, we also hypothesised that high stigma scores would be negatively associated with social support, and receipt of formal or informal support. To understand what differentiates those who attempt suicide from those who consider suicide after sudden bereavement, we hypothesised that high stigma scores are associated with suicide attempt in the sub-group of those with suicidal thoughts following bereavement [17]. Finally, we hypothesised that the effect of high stigma scores on primary outcomes would be modified by gender and by mode of bereavement, such that it would be more pronounced in women and in people bereaved by non-suicide causes.

## 2. Materials and Methods

### 2.1. Study Design

We analysed data from the UCL Bereavement Study [8,9]. This was a UK-wide cross-sectional survey of young adults aged 18–40 working and/or studying at UK higher education institutions (HEIs) who had experienced the sudden bereavement of a close friend or relative. This study had focused on young adults due to concerns about their risk of suicide [18] and the difficulties of engaging young suicidal men with services [19]. Full details of sampling for this closed online survey have been described elsewhere, including the survey instrument (see Supplementary Materials) [8,9]. Sampling via institution-wide email lists (to all staff and students) avoided the biases associated with recruiting a help-seeking sample, and was felt to be the most efficient, comprehensive and pragmatic means of recruiting a hard-to-reach population of young adults [20]. Of 5085 respondents to the survey, we included those who consented to participate, completed a stigma score, and specified their mode of bereavement (*n* = 3387). 

The study was approved by the UCL Research Ethics Committee in 2010 (ref: 1975/002). All participants provided online informed consent. 

### 2.2. Measures

Our exposure measure was high perceived stigma of the bereavement, defined using the 10-item stigma subscale of the Grief Experience Questionnaire (GEQ) [21]. The GEQ is a standardised, self-administered instrument for the assessment of the phenomenology of grief. It was originally developed in the U.S. using qualitative data from individuals bereaved by natural causes, accidental death, and suicide [22], and subsequently validated [21]. The stigma sub-scale includes items describing perceptions of others’ avoidance and lack of concern (see Box 1), capturing perceived rather than personal stigma. Responses to items in each subscale are rated using a 5-point Likert-style frequency scale, generating subscale scores of 5 to 25 (at 0.5 intervals). The majority of studies measuring GEQ scores use GEQ subscales rather than overall GEQ scores, allowing them to delineate specific components of grief [8,23,24,25]. Based on precedent [23] and the normal distribution of stigma scores in this sample, we used the mean to dichotomise stigma scores, classifying them as low (5 to 12) or high (12.5 to 25) to aid clinical interpretation.

Box 1 **GEQ stigma subscale items.**

**Stem:** Since the death how often did you….

1. feel like a social outcast?
2. feel like no-one cared to listen to you?
3. feel that neighbours and friends did not offer enough concern?
4. feel avoided by friends?
5. think people were gossiping about you or the person?
6. think that others didn’t want you to talk about the death?
7. feel somehow stigmatised by the death?
8. feel like people were probably wondering about what kind of personal problems you and the person had experienced?
9. think that people were uncomfortable offering their condolences to you?
10. feel like the death somehow reflected negatively on you or your family?

Our primary outcomes were self-reported suicidal ideation (“Have you ever thought of taking your life, even though you would not actually do it?”) [26] and self-reported suicide attempt (“Have you ever made an attempt to take your life, by taking an overdose of tablets or in some other way?”) [27] post-bereavement. These standardised, validated measures were derived from the Adult Psychiatric Morbidity Survey (APMS) [28], a regular national population survey in England, qualified by whether each was before or after the sudden bereavement, or both, to derive an incident measure. 

Our three secondary mental health outcomes were post-bereavement non-suicidal self-harm (self-poisoning and self-injury without suicidal intent) using the standardised, validated APMS measure [27] (adapted as above); post-bereavement suicidal and non-suicidal self-harm (aggregating the suicide attempt and non-suicidal self-harm measures, to correspond to that used in a major longitudinal study of self-harm in England [29]); and post-bereavement depression, using the Composite International Diagnostic Interview (CIDI) screen for lifetime depression [30], also validated for use in an online questionnaire [31] (adapted for incident cases as above).

Our three self-reported support measures were level of current social support (using a standardised ordinal measure from the APMS [28]); receipt of any formal bereavement support (using a binary measure developed for this study); and receipt of any informal bereavement support (using a binary measure developed for this study). Classification of formal and informal bereavement support was derived from similar British [32] and international [33] studies of service use . Self-help was excluded due to problematic categorisation in relation to formal versus informal bereavement support [34]. Thus, formal support was defined as that received from healthcare or social services staff; psychological therapists or counsellors; voluntary sector helplines or counsellors; police officers; funeral directors; coroners’ officers; teaching staff; school or HEI counselling services; line managers, or employer counselling services. Informal support was defined as that received from friends; family; spiritual/religious advisors, or complementary and alternative medicine practitioners.

We selected nine confounding variables on the basis of existing literature and clinical judgement: age; gender; socio-economic status (using the UK Office for National Statistics Standard Occupational Classification [35]); mode of sudden bereavement; kinship to the deceased; family history of suicide (excluding an index bereavement by suicide); pre-loss depression; pre-loss suicidal and non-suicidal self-harm; and years since sudden bereavement. Mode of bereavement was classified via self-report as bereavement by suicide, bereavement by sudden natural causes (e.g., cardiac arrest), and bereavement by sudden unnatural causes (e.g., accidental death). In the case of exposures to more than one mode of sudden bereavement, all those bereaved by suicide were classified as such, regardless of other exposures. Those bereaved by non-suicide death were asked to relate their responses to whichever person they had felt closest to, with exposure status classified accordingly. 

Missing data for model covariates and outcomes were less than 7%.

### 2.3. Statistical Analysis

We investigated simple associations between the outcome variables and exposure using χ2 tests or one-way analysis of variance, as appropriate.

We investigated the relationship between outcomes and high stigma scores using multilevel regression models with HEI as random effect, to take into account the clustering effect at the HEI level. We used ordinal logistic regression to investigate the relationship between social support and high levels of perceived stigma scores. All multivariable models included the nine pre-specified confounding variables described above. Models were fitted using complete case analysis. We used the Bonferroni correction to set a significance threshold of *p* = 0.006 for multiple testing.

To test whether the effect of high stigma scores on primary outcomes varied by gender and by mode of bereavement, we added interaction terms to these models, using a less stringent *p*-value threshold (*p* = 0.1) to reflect the limited statistical power of interaction tests.

To test an additional research question about whether high perceived stigma helps differentiate those who attempt suicide after bereavement from those with suicidal ideation after bereavement, we ran our multivariable model for suicide attempt in the sub-sample of those who reported suicidal thoughts or attempts post-bereavement (*n* = 1510). 

We ran a series of a priori defined sensitivity analyses to assess the robustness of our main findings when taking into account biases introduced by <7% missing data and by our sampling strategy. In the first and second analyses, we used best-case and worst-case scenarios to impute missing values by recoding all missing values on outcomes/covariates as positive (e.g., no suicidal ideation/attempt) or as negative (e.g., suicidal ideation/attempt) respectively [36]. In the third and fourth, we used more stringent inclusion criteria: dropping the 10 HEIs that modified the stipulated recruitment method, and the 18 HEIs with participant numbers below the median cluster size. Finally, we conducted linear regression to test whether there was a linear association between stigma scores and outcomes.

All analyses were conducted using Stata version 12 (Stata Corp. 2011. Stata Statistical Software: Release 12. College Station, TX, USA).

## 3. Results

### 3.1. Participant Characteristics

The majority of the sample were female (81%), of white ethnicity (90%), bereaved by sudden natural causes (61%), and reported the death of a relative (71%). The mean time elapsed since bereavement was 5 years (standard deviation (SD) = 5.3 years; range = 1 day to 30 years), with no group differences (Table 1). The age of the deceased varied from 0 (for miscarriage or stillbirth) to 100 years, and median age was significantly younger for those reporting high (median age = 45; inter-quartile range (IQR) = 22–58) versus low stigma scores (median = 50; IQR = 23–70). The group reporting high stigma scores were more likely to be women, students, those in higher social classes, and those educated to a higher level than the group with low stigma scores. They were also significantly more likely to have been bereaved by suicide, and to have had a history of suicidal or non-suicidal self-harm and of depression prior to the loss. 

Amongst subjects who had made a suicide attempt since the bereavement, those who reported high stigma scores were significantly less likely to have sought help for it than those reporting low stigma scores.

Overall, 32% of the sample had received no informal support after the bereavement (Table 2).

### 3.2. Association between High Stigma Scores and Outcomes

In an adjusted analysis (Table 3), high stigma scores were associated with a significantly higher probability of post-bereavement suicidal thoughts (AOR = 2.74; 95% CI = 1.93–3.89), suicide attempt (AOR = 2.73; 95% CI = 2.33–3.18), non-suicidal self-harm (2.16; 95% CI = 1.76–2.64), any self-harm (AOR = 2.25; 95% CI = 1.85–2.74), depression (AOR = 3.84; 95% CI = 3.21–4.59).

High stigma scores were positively associated with poor social support (AOR = 2.86; 95% CI = 2.44–3.34), and use of formal bereavement support (AOR = 1.87; 95% CI = 1.60–2.19), but negatively associated with use of informal bereavement support (AOR = 0.48; 95% CI = 0.41–0.57).

In the sub-sample of *n* = 1510 individuals who reported suicidal thoughts or attempts post-bereavement, we found a significant association between high stigma scores and post-bereavement suicide attempt (AOR = 1.90; 95% CI = 1.32–2.72; *p* = 0.001).

### 3.3. Interactions

Gender did not modify the associations between stigma and primary outcomes, but there was an interaction with mode of bereavement, such that the magnitude of the association between high stigma and suicidal ideation was higher for those bereaved by sudden natural death (AOR = 3.08; 95% CI = 2.52–3.76) or sudden unnatural death (AOR = 3.02; 95% CI = 2.13–4.28) than for those bereaved by suicide (AOR = 1.61; 95% CI = 1.09–2.39).

### 3.4. Sensitivity Analysis

The magnitude and direction of adjusted odds ratios for primary outcomes were unchanged in four sensitivity analyses simulating potential biases introduced by missing data and by our sampling strategy. Conducting the analysis using linear regression showed that stigma scores were significantly associated with suicidal ideation (adjusted coefficient = 0.033; 95% CI = 0.029–0.037; *p* ≤ 0.001) and suicide attempt (adjusted coefficient = 0.009; 95% CI = 0.006–0.107; *p* ≤ 0.001). On all other measures, it also showed directions of associations consistent with those in our main analysis (*p* ≤ 0.001 in all cases).

## 4. Discussion

### 4.1. Main Findings

The findings of this analysis of British cross-sectional data support our hypothesis that people who feel highly stigmatised by the sudden death of a friend or relative are at increased risk of suicidal thoughts, suicide attempt, non-suicidal self-harm, and depression. The cross-sectional nature of the data limits interpretation of the chronology of the pathways between high stigma scores and outcomes. However, associations with support measures suggested a buffering effect of social support on the negative effects of perceived stigma: those with low perceived stigma scores were more likely to report use of informal support, whereas those with high stigma scores perceived poor social support. Contrary to our hypothesis, use of formal support was more likely in people who felt highly stigmatised, perhaps because they could not rely on friends and family. It is possible that informal support plays an important role in preventing and/or redressing perceived stigma and in mitigating the effects of stigma on suicidality and depression. The results of our interaction tests are interpretable in the context of previous findings from this dataset, namely the increased risk of suicide attempt in people bereaved by suicide [9]. Amongst those bereaved by non-suicide causes, it was those perceiving high levels of stigma who were much more likely to report suicidal thoughts. 

### 4.2. Results in the Context of Other Studies

Previous studies measuring the stigma of sudden bereavement have compared groups defined by cause of death [1,9], but none have explored whether stigma scores per se are associated with adverse outcomes. Instead, qualitative approaches have been used to describe the nature of the stigma experienced by people bereaved traumatically, and how the “death taboo” influences their and others’ avoidance of the topic [11]. Qualitative studies of the stigma perceived by people with mental illness identify the anticipation of negative consequences as a key theme in relation to help-seeking behaviour [10]. The same might apply after bereavement, particularly where the bereaved anticipate social awkwardness [15]. There is some evidence that the stigma attached to help-seeking for mental health problems [10] also applies to bereavement support groups [38], even despite social expectations for the bereaved to engage with support [39] so they can quickly “move on” with their grief [40,41,42].

### 4.3. Strengths and Limitations

We analysed data from a large, UK-wide sample of 3387 bereaved adults using a validated stigma measure. We tested specific hypotheses formulated on the basis of theory and clinical experience, and our models were adjusted for pre-selected potential confounders. Results were robust to sensitivity analysis simulating potential biases. We acknowledge the potential for male non-response bias, and selection bias of highly educated adults from HEIs. This, and the restricted 18–40 age range, suggest that this study’s findings may only be generalizable to highly-educated young women in the UK. All measures were potentially subject to recall bias. Although validated, our measure of lifetime depression was derived from a brief screening tool [30], and may have over- or under-estimated past depression where used as a potential confounder in multivariable models. Our measures of formal and informal support use were subjective, and represent both preferences and availability. Although the GEQ stigma subscale captures stigmatising aspects of the death specifically, perceived stigma may have been compounded by stigmatising depression or suicidal behaviour. As this was a cross-sectional study, it was not possible to ascertain the temporal sequence of outcomes, including whether suicidal behaviour following bereavement had preceded the awareness of stigma, or of lack of support. However, quantitative [43] and qualitative [44] studies identify perceptions of a lack of support immediately after a sudden death. 

### 4.4. Clinical and Policy Implications

This study has identified perceived stigma after sudden bereavement as a potentially useful marker for suicidality, depression, and for poor social support. Indeed, among those who had felt suicidal following sudden bereavement, perceptions of high stigma helped differentiate attempters from ideators. Identification of people who feel highly stigmatised after bereavement creates an opportunity to intervene and prevent suicide attempt. General practitioners and bereavement counsellors who encounter bereaved people might consider inquiring about perceived stigma as a way of building a rapport before probing, where appropriate, for low mood and thoughts of self-harm and suicide. Anyone in contact with someone bereaved traumatically has a role in providing information on sources of voluntary sector bereavement support [45,46,47]. Given our findings, this is particularly important for those who feel most stigmatised. Specific resources are available for people bereaved by suicide [46,48], recognising their elevated risk of suicide [2,4] and efforts to target this group in suicide prevention strategies [49,50]. 

### 4.5. Future Research

The role of stigma as a putative mediator in the association between sudden bereavement and suicide-related outcomes, and the role of informal support as a moderator of this effect, would need formal testing using longitudinal approaches. If stigma is confirmed as mediating risk of suicidality, the next step would be to develop and trial individual-level or community-level anti-stigma interventions. These might address the barriers to seeking or receiving support, and potentially reduce suicide rates. Cultural dimensions of grief [51] suggest that the development of anti-stigma interventions will need to be based on the findings of qualitative studies. These should explore why bereaved people in different communities feel stigmatised, and how this influences help-seeking behaviour and mental health, but also to understand how informal networks perceive their role and what prevents them from offering adequate support.

## 5. Conclusions

The results of this study suggest that people who feel the most stigmatised by a sudden bereavement are at greater risk of suicidal behaviour and depression, and are more likely to feel inadequately socially supported by friends and family. High perceived stigma helps differentiate those who attempt suicide after sudden bereavement from those who consider it. Clinicians who inquire about perceived stigma are in a position to identify suicidal distress and address support needs. All those who have contact with people bereaved traumatically should direct them to appropriate sources of support, to overcome any barriers to help-seeking and the effects of perceived stigma. 

## Figures and Tables

**Table 1:** Characteristics of participants by high versus low perceived stigma scores.

GEQ Stigma Sub-Scale Score	Low Perceived Stigma Score ^a^ (*n* = 1764)	High Perceived Stigma Score ^a^ (*n* = 1623)	Total (*n* = 3387)	*p*-Value ^b^
**Socio-demographic characteristics**				
**Gender** †				
Female *n* (%)	1388 (79)	1360 (84)	2748 (81)	<0.001
missing *n* (%)	0 (0)	1 (<1)	1 (<1)	
**Age of participant** †				
mean (SD)	25.0 (6.2)	25.1 (6.4)	25.0 (6.3)	1.000
**Self-defined ethnicity**				
white *n* (%)	1598 (91)	1449 (89)	3047 (90)	0.224
non-white *n* (%)	165 (9)	172 (11)	337 (10)	
missing *n* (%)	1 (<1)	2 (<1)	3 (<1)	
**Socio-economic status** ^c^ †				
social classes 1.1 and 1.2 *n* (%)	551 (31)	440 (27)	991 (29)	<0.001
social class 2 *n* (%)	586 (33)	526 (32)	1112 (33)	
social class 3 *n* (%)	209 (12)	185 (11)	394 (12)	
social class 4 *n* (%)	91 (5)	63 (4)	154 (5)	
social classes 5–7 and 9 *n* (%)	273 (16)	359 (22)	632 (19)	
missing *n* (%)	54 (3)	50 (3)	104 (3)	
**Educational status**				
attained up to A level equivalent leaving qualification *n* (%)	734 (42)	750 (46)	1484 (44)	0.008
attained undergraduate degree or above *n* (%)	1025 (58)	871 (54)	1896 (56)	
missing *n* (%)	5 (<1)	2 (<1)	7 (<1)	
**Student status**				
student *n* (%)	1472 (83)	1428 (88)	2900 (86)	0.001
staff *n* (%)	238 (13)	156 (10)	394 (12)	
both *n* (%)	54 (3)	38 (2)	92 (3)	
missing *n* (%)	1 (0)	1 (<1)	1 (<1)	
**Characteristics of index bereavement**				
**Mode of death**				
sudden natural causes *n* (%)	1184 (67)	892 (55)	2076 (61)	<0.001
sudden unnatural causes *n* (%)	368 (21)	336 (21)	704 (21)	
suicide *n* (%)	212 (12)	395 (24)	607 (18)	
missing *n* (%)	0 (0)	0 (0)	0 (0)	
**Kinship to the deceased** †				
blood relative *n* (%)	1224 (69)	1176 (73)	2400 (71)	0.050
unrelated *n* (%)	533 (30)	441 (27)	974 (29)	
missing *n* (%)	7 (<1)	6 (<1)	13 (<1)	
**Age of the deceased**				
median (IQR)	50 (23–70)	45 (22–58)	47 (23–64)	<0.001
**Gender of the deceased**				
Female *n* (%)	666 (38)	583 (36)	1249 (37)	0.216
missing *n* (%)	54 (3)	41 (3)	95 (3)	
**Time since bereavement** †				
mean (SD)	4.6 (5)	5.4 (6)	5 (5.3)	1.000
**Clinical characteristics**				
**Family history of suicide (excluding index suicide bereavement)** †				
Yes *n* (%)	109 (6)	104 (6)	213 (6)	0.829
missing *n* (%)	128 (7)	106 (7)	234 (7)	
**Family history of psychiatric problems**				
Yes *n* (%)	1008 (57)	1059 (65)	2067 (61)	<0.001
missing *n* (%)	124 (7)	102 (6)	226 (7)	
**Personality disorder screen positive** ^d^				
Yes *n* (%)	464 (26)	712 (44)	1176 (35)	<0.001
missing *n* (%)	105 (6)	84 (5)	189 (6)	
**Pre-loss depression** †				
Yes *n* (%)	280 (16)	353 (22)	633 (19)	<0.001
missing *n* (%)	74 (4)	54 (3)	128 (4)	
**Pre-loss non-suicidal self-harm and suicide attempt** †				
Yes *n* (%)	325 (18)	380 (23)	705 (21)	<0.001
missing *n* (%)	127 (7)	104 (6)	231 (7)	
**Help sought after suicide attempt post-bereavement**				
Yes *n* (%)	15 (1)	54 (3)	69 (2)	<0.001
No *n* (%)	36 (2)	101 (6)	137 (4)	
No suicide attempt post-bereavement *n* (%)	1713 (97)	1468 (91)	3181 (94)	

SD = standard deviation; IQR = inter-quartile range; ^a^ using Grief Experience Questionnaire (GEQ) stigma sub-scale score dichotomised at mean into low (5 to 12) and high (12.5 to 25). † pre-specified covariate entered into adjusted models. ^b^ significance threshold of *p* = 0.05; not adjusted for multiple testing. ^c^ socio-economic status using the five categories from UK Office for National Statistics. ^d^ SAPAS-SR screen for personality disorder [37].

**Table 2:** Summary of outcomes by low versus high perceived stigma scores.

GEQ Stigma sub-Scale Score	Low Perceived Stigma Score ^a^ (*n* = 1764)	High Perceived Stigma Score ^a^ (*n* = 1623)	Total (*n* = 3387)	*p*-Value ^b^
**Primary outcomes**				
**Post-loss suicidal thoughts**				
Yes *n* (%)	582 (33)	929 (57)	1511 (45)	<0.001
missing *n* (%)	121 (7)	100 (6)	221 (7)	
**Post-loss suicide attempt**				
Yes *n* (%)	51 (3)	155 (10)	206 (6)	<0.001
missing *n* (%)	126 (7)	100 (6)	226 (7)	
**Secondary outcomes**				
**Secondary mental health outcomes**				
**Post-loss non-suicidal self-harm**				
Yes *n* (%)	260 (15)	473 (29)	733 (22)	<0.001
missing *n* (%)	125 (15)	102 (6)	227 (7)	
**Post-loss non-suicidal self-harm and suicide attempt**				
Yes *n* (%)	280 (16)	519 (32)	799 (24)	<0.001
missing *n* (%)	121 (7)	98 (6)	219 (7)	
**Post-loss depression**				
Yes *n* (%)	361 (21)	699 (43)	1060 (4)	<0.001
missing *n* (%)	74 (4)	54 (3)	128 (4)	
**Support measures**				
**Measure of social support** ^c^				
no lack of perceived social support *n* (%)	1228 (70)	739 (46)	1967 (58)	<0.001
moderate lack of perceived social support *n* (%)	406 (23)	499 (31)	905 (27)	
severe lack of perceived social support *n* (%)	130 (7)	384 (24)	514 (15)	
missing *n* (%)	0 (0)	1 (<1)	1 (<1)	
**Receipt of formal support after index bereavement**				
Yes *n* (%)	553 (31)	704 (43)	1257 (37)	<0.001
No *n* (%)	1135 (64)	861 (53)	1996 (59)	
missing *n* (%)	76 (4)	58 (4)	134 (4)	
**Receipt of informal support after index bereavement**				
Yes *n* (%)	1277 (72)	907 (56)	2184 (65)	<0.001
No *n* (%)	411 (23)	658 (41)	1069 (32)	
missing *n* (%)	76 (4)	58 (4)	134 (4)	

GEQ = Grief Experience Questionnaire, a using GEQ stigma sub-scale score. ^a^ using Grief Experience Questionnaire stigma sub-scale score dichotomised at mean into low (5 to 12) and high (12.5 to 25). ^b^ significance threshold of *p* = 0.05; not adjusted for multiple testing. ^c^ measure of social support from Adult Psychiatric Morbidity Survey [28].

**Table 3:** Estimates of the association between high stigma scores and outcomes.

GEQ Stigma Sub-Scale Score	Low Perceived Stigma Score ^a^ (*n* = 1764)	High Perceived Stigma Score ^a^ (*n* = 1623)			
Primary Outcomes	Odds Ratio (reference)	Unadjusted Odds Ratio (95% CI)	*p* Value ^b^	Adjusted ^c^ Odds Ratio (95% CI)	*p* Value ^b^
post-bereavement suicidal ideation	1	3.45 (2.47–4.81)	<0.001	2.74 (1.93–3.89)	<0.001
post-bereavement suicide attempt	1	2.84 (2.45–3.89)	<0.001	2.73 (2.33–3.18)	<0.001
**Secondary Measures**					
post-bereavement non-suicidal self-harm	1	2.40 (2.01–2.86)	<0.001	2.16 (1.76–2.64)	<0.001
post-bereavement suicidal and non-suicidal self-harm	1	2.50 (2.11–2.96)	<0.001	2.25 (1.85–2.74)	<0.001
post-bereavement depression	1	2.91 (2.48–3.41)	<0.001	3.84 (3.21–4.59)	<0.001
low perceived social support	1	2.84 (2.45–3.28)	<0.001	2.86 (2.44–3.34)	<0.001
use of formal bereavement support	1	1.76 (1.52–2.04)	<0.001	1.87 (1.60–2.19)	<0.001
use of informal bereavement support	1	0.44 (0.37–0.51)	<0.001	0.48 (0.41–0.57)	<0.001

GEQ = Grief Experience Questionnaire. ^a^ using Grief Experience Questionnaire stigma sub-scale score dichotomised at mean into low (5 to 12) and high (12.5 to 25). ^b^ using corrected significance threshold of *p* = 0.006. ^c^ adjusted for nine pre-specified confounding variables: age; gender; socio-economic status; mode of sudden bereavement; kinship to the deceased; family history of suicide (excluding index bereavement); pre-loss depression; pre-loss suicidal and non-suicidal self-harm; and years since index bereavement.

## Supplementary Materials

The following are available online at www.mdpi.com/1660-4601/14/3/286/s1.
